# A review of optimal evaluation and treatment of suspected esophageal food impaction

**DOI:** 10.1007/s10140-020-01855-5

**Published:** 2020-10-27

**Authors:** MeNore Lake, David Smoot, Peter O’Halloran, Michael Shortsleeve

**Affiliations:** Mount Auburn Hospital, 330 Mount Auburn Street, Cambridge, MA 02138 USA

**Keywords:** Acute esophageal food impaction, Esophageal disimpaction, Fluoroscopy, Endoscopy, Glucagon, Combination therapy, Schatzki ring

## Abstract

Fluoroscopy-guided esophageal disimpaction of ingested food is a safe, effective, and cost-efficient alternative to endoscopically guided disimpaction. Patients with suspected esophageal impaction usually require fluoroscopy to confirm the diagnosis and determine the level of obstruction, which guides further management. Proximal esophageal food impactions at or near the cricopharyngeus muscle require an ENT intervention. Food impactions from the cervical esophagus to the aortic arch require a GI intervention. Obstructions distal to the aortic arch can usually be managed by the radiologist with a fluoroscopy-guided disimpaction. The use of intravenous glucagon to relax the mid and distal esophageal smooth muscle, combined with an effervescent agent, and water comprises this “combination” therapy to relieve an acute esophageal food impaction. This paper reviews the indications, contraindications, technique, and 32 years of experience with fluoroscopy-guided esophageal disimpaction at our institution. A retrospective chart review of our experience includes 252 patients with a 56% success rate that obviated more expensive and invasive procedures. Only one complication of a minor mucosal tear of no clinical consequence was encountered. Radiologists should be familiar with the presentation and management of this common diagnosis.

## Introduction

**Fig 1:**
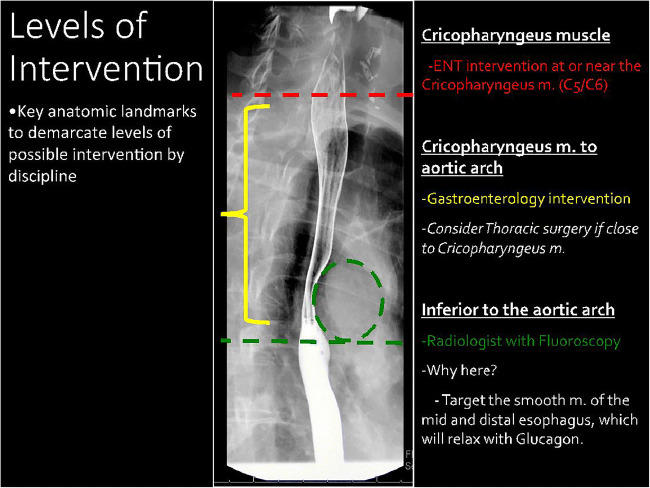
Levels of intervention

Esophageal food impaction is a common presentation to the emergency department with various treatment algorithms based on institution and availability of ear, nose, and throat (ENT) and gastroenterological (GI) specialties. Patients with suspected esophageal food impactions usually require imaging in fluoroscopy to confirm the diagnosis and determine the level of obstruction, which guides further management. The emergency department physician uses the fluoroscopy findings to choose which service to consult for further management. For example, a proximal obstruction at or near the cricopharyngeus muscle requires the intervention of ENT specialists. Alternatively, if the obstruction is located from the cervical esophagus to the aortic arch, then the GI service is consulted for possible flexible endoscopic retrieval. For impactions close to the cricopharyngeus muscle, thoracic surgery consultation may be necessary. This paper outlines the management of obstructions of the esophagus distal to the aortic arch by the radiologist with a fluoroscopy-guided disimpaction; an anatomic diagram demonstrating these levels of intervention is demonstrated in Fig. 1.

This simple procedure utilizes glucagon to relax the mid and distal esophageal smooth muscle and an effervescent agent to distend the relaxed esophagus combined with prompt drinking of water to increase the hydrostatic pressure above the bolus. This technique is referred to as “combination” therapy and is a safe approach to dislodge the impacted food bolus. The purpose of this article is to review the indications, contraindications, technique, and experience with fluoroscopic-guided esophageal disimpaction at our institution over the last 32 years.

## Materials and methods

### Data collection

An Institutional Review Board approval was granted to retrospectively review 32 years of experience at our facility spanning from 1987 through 2019. Various techniques were required to identify cases over the years due to changes in coding and availability of the medical record prior to the introduction of digital PACS. The patients ranged from 17 years old to 95 years old (76 females, 176 males).

Cases from 1987 through 1993 and between 2001 and 2002 were not available on digital or physical record in the hospital, so the previous departmental publication from 1994 introducing fluoroscopy-guided esophageal disimpaction was referenced and yielded 48 cases [1, 2].

Cases from 1993 through 1997 were not available on digital or physical record and were not previously published; therefore, these cases were not available for review.

Cases from 1997 through 2001 were identified through the radiology department PACs exam code (BAS), yielding 3 cases.

Cases from 2002 to 2013 were identified by tracking the radiology department’s use of glucagon, which is one of the agents used in this procedure, and yielded 110 cases for review (Shortsleeve, unpublished data).

Cases from 2013 through 2019 were identified through the radiology department PACS by searching for the appropriate exam code (IMG742), yielding 91 cases from 2013 to 2019.

### Indications/contraindications

Fluoroscopy-guided disimpaction is indicated in patients with acute food impaction in the distal two-thirds of the esophagus.

Contraindications for intervention can be categorized by parameters elicited through clinical history and by parameters demonstrated on the initial fluoroscopic examination, as illustrated in Table 1. Contraindications include impaction with a sharp foreign body (e.g., plastic eating utensils and bones, which increase perforation risk) and symptoms for over 24 hours (as beyond this time period, it is more likely that the food impaction has eroded the mucosa of the esophagus, predisposing the patient to increased perforation risk). History of esophageal manipulation, including esophageal dilation or esophagogastroduodenoscopy (EGD) within the past 7 days, is also a contraindication to this intervention, as these manipulations predispose the esophagus to perforation.

**Table 1:** Contraindications for intervention

Based on history	• > 24-hours duration • Caused by a known sharp foreign body ○ Ex: bone, plastic eating utensil ○ Also evaluated on initial fluoroscopic evaluation • Known esophageal stricture (benign or malignant)-strictures do not relax with glucagon • Esophageal manipulation in the last week ○ Ex: EGD, esophageal dilation • Contraindications to glucagon ○ Anaphylaxis to glucagon ○ Insulinoma or pheochromocytoma ○ Use with caution in diabetes mellitus (theoretical risk of hyperglycemia particularly in type 1 diabetes mellitus)
Based on the initial fluoroscopic exam	• Obstruction in the proximal third of the esophagus, which is striated skeletal muscle • Prominent cricopharyngeus m. (includes if previously described) • Zenker’s diverticulum • A diverticulum of the mid or distal esophagus

Contraindications to glucagon such as anaphylaxis to glucagon or the diagnosis of pheochromocytoma or insulinoma also preclude fluoroscopic intervention. Glucagon is contraindicated in the setting of pheochromocytoma because glucagon stimulates catecholamine release, increasing the risk of inducing a sudden state of marked hypertension. In a patient with an insulinoma, glucagon is contraindicated due to the risk of inducing rebound hypoglycemia. Although the diagnosis of diabetes mellitus is not a contraindication to combination therapy, the care team should be aware of glucagon’s theoretical risk of inducing hyperglycemia.

Specific anatomic features of the esophagus can also be a contraindication for fluoroscopic intervention. For example, the presence of esophageal stricture, benign, or malignant, is a contraindication for intervention. While treating a patient with a stricture would not harm the patient, these cases often fail because the stricture cannot relax with glucagon or distend with the effervescent agent. As a community teaching hospital, we commonly have seen our patients in the past and have access to their medical records; rather than intervention in these scenarios of a known stricture, we refer directly to the appropriate specialist.

A prominent cricopharyngeus muscle or an esophageal diverticulum, either noted on prior studies or evident upon the initial fluoroscopic evaluation, is also a contraindication for combination therapy. In these contexts, the prominent muscle would obstruct the upward release of the ingested carbon dioxide gas. It is specifically clarified that, as a Schatzki ring is not an esophageal stricture, it is thus not a contraindication for intervention; the presence of a Schatzki ring is a common feature of our candidate population, and this sub-population tends to respond well to the combination therapy.

### The procedure

The radiologist must follow the standard radiation safety protocol of wearing protective eyewear and a lead apron.

**Fig. 2:**
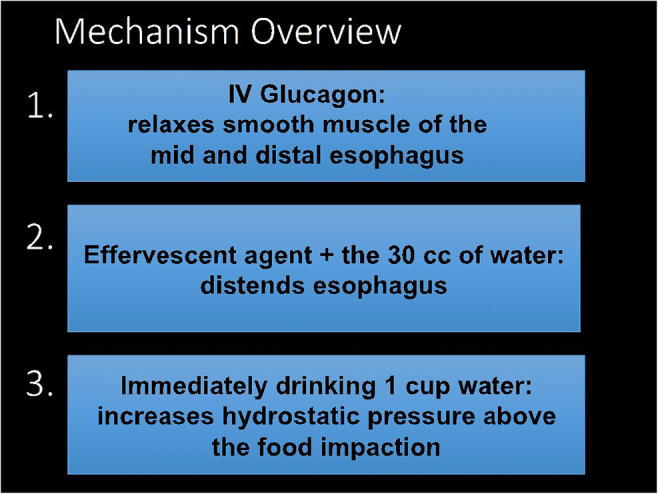
Mechanism overview. This technique is referred to as “combination therapy,” due to the combined effects of its key reagents (IV glucagon, effervescent agent, and water) in relieving an acute esophageal food impaction

**Fig. 3:**
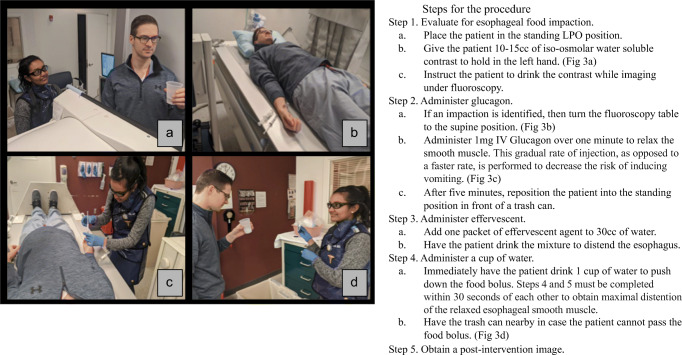
Steps for the procedure

The three defining mechanisms of combination therapy are demonstrated in Fig. 2. Steps 1 through 5 describe the technique of the esophageal disimpaction procedure and are illustrated in Fig. 3.

**Fig. 4:**
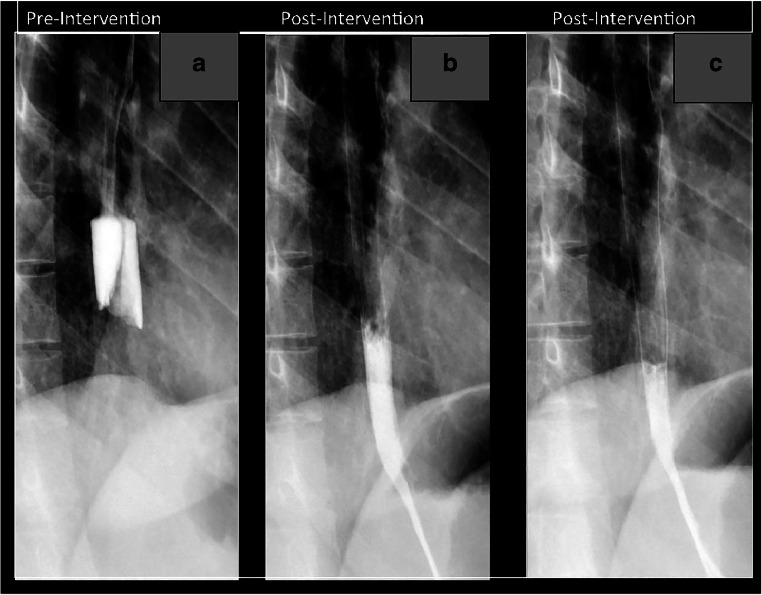
Acute esophageal food impaction, pre and immediate post intervention. The pre intervention image (**a**) shows an impacted food bolus in the distal two-thirds of the esophagus, with the patients in the standing LPO position. The post intervention images (**b**, **c**) were obtained immediately after completion of the disimpaction protocol. The food impaction is gone, as evidenced by the new transit of contrast, and there is no esophageal leak in the immediate post-intervention images. Each of the images was obtained shortly after the patient drank iso-osmolar, water-soluble contrast

Step 1 Evaluate for esophageal food impaction:
Place the patient in the standing left posterior oblique position (LPO).
Give the patient 10–15 cc of iso-osmolar, water-soluble contrast. See Fig. 4, image A for components 1 and 2 of this step.
Instruct the patient to drink the 10–15 cc of iso-osmolar water-soluble contrast.
Observe the contrast bolus passing down the esophagus.

Step 2 If there is a mid or distal esophageal impaction identified and the patient does not have any contraindications to fluoroscopically guided intervention, then glucagon can be administered.
1) Turn the fluoroscopic table to the horizontal position (patient should be supine).
2) Over a period of 1 minute, administer 1 mg IV glucagon. This gradual rate of injection, as opposed to a faster rate, is performed to decrease the risk of inducing vomiting.

Step 3 After waiting for 5 minutes for the glucagon to relax the esophageal smooth muscle, re-position the patient into the standing position next to the fluoroscope with a trash can between the patient and radiologist in case of emesis.
Step 4 Add 1 packet of the effervescent agent to 30 ml of water and drink.
Step 5 Promptly drink 1 cup of water. Steps 4 and 5 must be completed within 30 seconds of each other to obtain maximal distention of the relaxed esophageal smooth muscle.

The LPO position optimizes the evaluation of the esophagus, as it is offset from the spine in this projection.

There are two critical features of the contrast used in this procedure. First, the contrast must be iso-osmolar; in this scenario, if the patient aspirates the contrast, pulmonary edema is avoided. Second, the contrast must be water soluble. If the patient has an esophageal perforation, water-soluble contrast, rather than barium, would leak into the mediastinum; thus, this avoids the risk of barium-associated mediastinitis if there is a perforation. Additionally, if endoscopic gastroduodenoscopy (EGD) is subsequently performed, the gastroenterologist will be able to see through the transparent, water-soluble contrast from the fluoroscopic exam.

Observing the contrast bolus passing down the esophagus demonstrates the presence or absence of impaction and the level of obstruction. Impactions below the level of the aortic arch involve the smooth musculature of the mid and distal esophagus, which will relax with glucagon.

The effervescent agent functions to distend the relaxed esophagus, and the prompt drinking of water increases the hydrostatic pressure above the food bolus; the combined effect is usually able to relieve an acute esophageal food impaction.

### A unique patient scenario: the diagnostic-only exam

Our department has encountered many patients that present with a suspected esophageal impaction but are not candidates for therapeutic intervention by fluoroscopy due to at least one contraindication. The most common scenario is that of a patient who has experienced obstructive symptoms for greater than 24 hours.

In this scenario, the protocol should be tailored to prioritize patient safety and still provide valuable diagnostic information to our referring clinicians. The procedure must be abridged, stopping after step 1; with this shortened protocol, the fundamental questions of “is there an obstruction” and “at what level is the obstruction” can be answered. This diagnostic-only exam can guide the management plan for the patient, including possible ENT versus GI consultation.

### Immediate post procedure evaluation

After the full combination therapy protocol is completed, an immediate post-intervention fluoroscopic image is obtained. The immediate study is performed with water-soluble contrast and answers two critical questions: did the impacted food pass and is there an esophageal leak? After both successful and unsuccessful cases, 20 cc of iso-osmolar, water-soluble contrast per swallow is given to the patient for this assessment. In cases of a successful disimpaction, two to three swallows are observed, while in an unsuccessful case, only one swallow is observed for this post-procedure evaluation. The immediate post intervention evaluation is demonstrated in Fig. 4.

## Results

Over the past 32 years at our institution, 252 patients were identified who have been treated with the fluoroscopic-guided esophageal disimpaction, resulting in a 56% success rate (140 of 252 patients). These 140 successful disimpactions obviated more expensive and invasive procedures. The time elapsed between the inciting event to this fluoroscopic intervention varied; however, the full intervention was only performed if the time elapsed was less than 24 hours and if the patient did not have any other contraindication, per protocol. Only one complication of a minor mucosal tear of no clinical consequence was encountered after two repeated failed attempts at fluoroscopic esophageal disimpaction. The minor mucosal tear was identified on follow-up endoscopy; however, the patient was asymptomatic and the tear healed without further intervention. After this complication, the protocol was modified to clarify that only one attempt should be made per episode of impaction. Since this modification to the protocol in 1987, no complications have been encountered.

## Discussion

Our review of fluoroscopic esophageal disimpaction reaffirms the findings of prior studies: this combination technique is effective and safe as initial management for the appropriate patient. The vast majority of patients are ultimately found to have a pre-existing condition; the most common pre-existing condition is a Schatzki ring, which responds very well to our combination therapy. The single complication of a minor mucosal tear occurred after two unsuccessful attempts, one immediately after the other. An EGD performed later on the same day detected this mucosal tear. A one-attempt-only policy for this procedure was subsequently implemented in 1987, and no complications have occurred in the 32 years since its installment.

This procedure also represents a cost-effective option. The technical charge of the fluoroscopic evaluation for the patient is less than the cost of an EGD; the $384 versus $1680 price points from a case at our institution in June 2019 are demonstrated in Figs. 6.

**Fig. 5:**
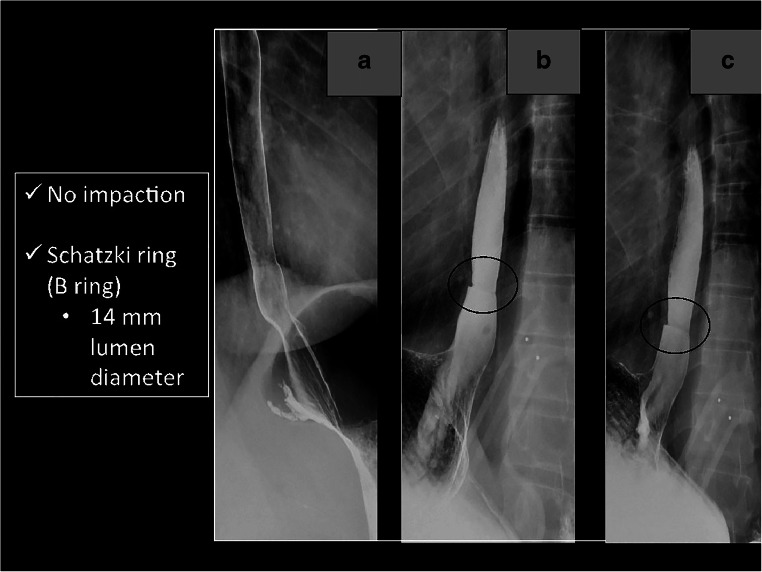
Two-month follow-up esophagram. This is a 2-month follow-up exam of the same patient from Fig. 4. Barium contrast passes through the esophagus without obstruction (**a**). Images **b** and **c** demonstrate a Schatzki ring with a 14-mm lumen diameter (circle). The presence of a Schatzki ring is not a contraindication in a patient presenting with acute esophageal food impaction, as this is not a stricture

**Fig. 6:**
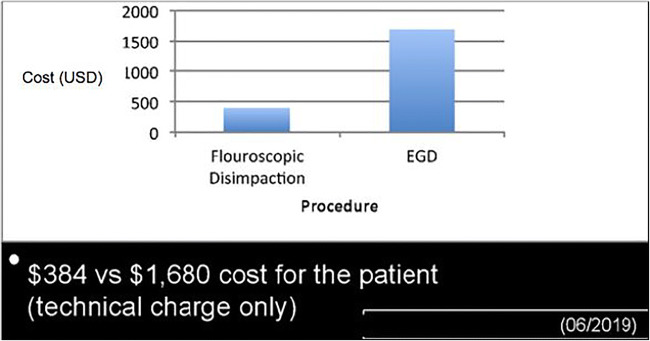
Cost profiles: fluoroscopic disimpaction vs EGD as initial management

In cases of a successful fluoroscopic esophageal disimpaction, our institution’s protocol is to recommend a follow-up study in approximately 2 weeks. This permits assessment for an underlying structural abnormality as the cause of the patient’s obstruction. A full esophagram is conducted on follow-up, with the benefit of resolution of the acute edema of the esophagus from the patient’s acute presentation. Fig. 4 is an example of a successful disimpaction case at our institution; this patient’s formal follow-up barium esophagram, which was 2 months later, is shown in Fig. 5.

The contraindications to performing the full esophageal disimpaction protocol serve as limitations to this approach, as a patient with at least one contraindication precludes completion of the therapeutic component of the procedure. This procedure also requires that the patient has an intact gag reflex; patients that lack this reflex should not receive combination therapy, as they are at increased risk of aspiration.

## Conclusion

Radiologists should be familiar with the diagnosis and management of acute esophageal food impaction, a common diagnosis. Fluoroscopically guided esophageal disimpaction should be the first line of therapy offered for appropriate patients with mid and distal esophageal food impaction due to ease of administration, low radiation dose, cost savings, and excellent safety profile.
